# Effects of Feeding Regime and Finishing Duration on Growth Performance, Meat Quality, Rumen Fermentation, and Microbiota in Black Angus Bulls

**DOI:** 10.3390/vetsci13070672

**Published:** 2026-07-10

**Authors:** Lijun Shang, Guanzhu Liu, Shuang Wu, Yan Ren, Bo Liu, Yunzhou Wang, Tao Wang, Lianyu Yang

**Affiliations:** 1 College of Animal Science and Technology, Jilin Agricultural University, Changchun 130118, China; 2 Jilin Province Changchun Haoyue Halal Meat Co., Ltd., Changchun 130118, China; 3 Liaoning Agricultural Vocational and Technical College, Yingkou 115009, China; 4 COFCO Feed Co., Ltd., Tangshan 064104, China

**Keywords:** Black Angus bull, corn silage, finishing duration, feeding regimes

## Abstract

Optimizing feeding regimes and finishing duration is essential for improving beef production efficiency and meat quality. This study evaluated the effects of two feeding regimes and three finishing durations on growth performance, nutrient digestibility, carcass traits, meat quality, blood biochemical parameters, rumen fermentation, and rumen microbiota in Black Angus bulls. A total of 552 healthy bulls with similar initial body weight were assigned to a 2 × 3 factorial design with two diets—a corn silage–rice straw–concentrate TMR (CSRC) and a rice straw–concentrate TMR (RSC)—and three finishing durations: 10, 12, and 14 months. The CSRC TMR increased average daily gain and reduced feed-to-gain ratio during early finishing (*p* < 0.05), whereas the RSC TMR resulted in greater final body weight and higher meat redness at 14 months (*p* < 0.05). Prolonged finishing decreased average daily gain, feed efficiency and nutrient digestibility (*p* < 0.05), but increased carcass weight, eye muscle area, backfat thickness and meat tenderness (*p* < 0.05). The CSRC TMR promoted higher total volatile fatty acid production (*p* < 0.05), an acetate-oriented fermentation pattern and greater rumen microbial alpha diversity. These findings support a stage-specific feeding regime for Black Angus bull finishing. Under the present conditions, corn silage inclusion may be used to improve early finishing efficiency, whereas rice straw–concentrate TMR may be more suitable for late finishing to balance final body weight, meat quality, and production cost.

## Simple Summary

Beef producers need practical ways to use local feed resources while producing good-quality beef at reasonable cost. In areas where corn silage and rice straw are available, it is unclear which feeding regime is more suitable at different fattening stages. This study compared 552 Black Angus bulls fed either a total mixed ration (TMR), containing corn silage, rice straw, and grain-based concentrate feed, or a TMR, containing rice straw and grain-based concentrate feed, for 10, 12 or 14 months. The TMR containing corn silage helped bulls gain weight more efficiently during the early fattening period. With longer fattening, bulls became heavier and the beef became redder and more tender, but animals needed more feed for each unit of weight gain and the cost of producing meat increased. The rice straw and concentrate TMR performed better during the later fattening period in terms of final body weight and meat redness. The results suggest that farms should adjust feeding regimes according to fattening stage, which may improve beef production efficiency, meat quality and economic return.

## 1. Introduction

High-quality beef production has become increasingly important with the rising demand for premium animal products. Black Angus bulls are widely recognized for their favorable carcass characteristics, meat quality, and potential for intramuscular fat deposition [1,2]. However, in commercial Angus finishing systems, economic return is determined not only by genetic potential but also by feeding strategy, local roughage availability, finishing duration, and the balance between feed efficiency, carcass value, and meat quality [3,4].

In Northeast China and other grain-producing regions, whole-plant corn silage and crop residues such as rice straw are important roughage resources for beef cattle [5,6]. Whole-plant corn silage is an important roughage source for ruminants because it provides digestible fiber, fermentable carbohydrates, organic acids, and good palatability, thereby supporting rumen fermentation and microbial activity [7,8]. In contrast, rice straw is inexpensive and widely available, and its physically effective fiber can stimulate chewing and rumination, helping to maintain rumen function in concentrate-based feeding systems [9,10]. However, its feeding value is limited by low digestibility and poor nutrient availability, mainly due to its high fiber and lignin contents [10,11]. Therefore, the optimal use of corn silage and rice straw in Angus bulls finishing remains a practical and scientific question.

Finishing duration is another key determinant of beef production efficiency and carcass value [4]. Extending the finishing period generally increases final body weight, hot carcass weight, carcass fatness, and the likelihood of achieving higher-quality grades [12]; however, prolonged finishing may reduce average daily gain and feed efficiency because older and heavier bulls allocate a greater proportion of nutrient intake to maintenance and fat deposition rather than lean tissue accretion [13]. Therefore, excessive finishing may increase production cost and reduce economic efficiency [4]. Determining the appropriate finishing endpoint is especially important for high-quality beef production systems in which both carcass value and feed cost must be considered [14].

The rumen is the central site of microbial fermentation and nutrient conversion in ruminants, where dietary carbohydrates are fermented into volatile fatty acids and microbial biomass [15,16]. Different roughage sources and dietary carbohydrate profiles can alter rumen fermentation patterns and reshape rumen microbial communities, thereby influencing nutrient digestion, energy supply, and growth performance [7,17]. Volatile fatty acids, particularly acetate and propionate, are major energy substrates for bulls; acetate is closely associated with fiber fermentation and lipid synthesis, whereas propionate is the major glucogenic precursor [15,18]. Therefore, integrating production performance with rumen fermentation and rumen microbiota may help clarify how feeding regime and finishing duration regulate the performance of Angus bulls.

Although previous studies have investigated the effects of dietary energy level, roughage source, or finishing duration on beef cattle production [4,17,19], most have considered these factors separately. Limited information is available on whether feeding regime and finishing duration interact to regulate growth efficiency, meat quality, rumen fermentation, and rumen microbial adaptation in Black Angus bulls. In particular, the stage-specific value of corn silage–rice hay–concentrate and rice hay–concentrate feeding regimes remains unclear. The novelty of this study lies in the simultaneous evaluation of two feeding regimes and three finishing durations using a large-scale 2 × 3 factorial design, together with an integrated assessment of growth performance, apparent total-tract nutrient digestibility, carcass traits, meat quality, serum biochemical parameters, rumen fermentation, and rumen microbial composition.

We hypothesized that feeding regime and finishing duration would interact to regulate growth efficiency, meat quality, rumen fermentation, and rumen microbiota in Black Angus bulls. Therefore, this study aimed to evaluate the effects of two feeding regimes and three finishing durations on growth performance, apparent total-tract nutrient digestibility, carcass traits, meat quality, serum biochemical parameters, rumen fermentation, and rumen microbial composition in Black Angus bulls.

## 2. Materials and Methods

All animal procedures were approved by the Institutional Animal Care and Use Committee at Jilin Agriculture University (2024 03 15 011). During the trial, animals were managed under commercial beef cattle production conditions, with routine health management, vaccination, deworming, free access to drinking water, and regular cleaning and disinfection of housing facilities.

### 2.1. Animals, Experimental Design, and Management

A total of 552 healthy Black Angus bulls aged 7–8 months, with a similar initial body weight of 246.01 ± 3.26 kg, were used in this study. The feeding trial was conducted at a commercial beef cattle farm in Nong’an County, Changchun, Jilin Province, China.

The experiment used a completely randomized 2 × 3 factorial design. The two factors were feeding regime and finishing duration. The two feeding regimes were: (1) corn silage–rice straw–concentrate total mixed ration (TMR), abbreviated as CSRC; (2) rice straw–concentrate TMR, abbreviated as RSC. The three finishing durations were 10 months (T10), 12 months (T12), and 14 months (T14). The 552 bulls were first divided into two feeding groups, with 276 animals per feeding regime. Within each feeding regime, animals were further divided into three finishing-duration groups, with 92 animals per treatment combination.

Animals were housed in comparable pens, with 5–6 bulls per pen. After entering the trial, all bulls underwent routine deworming and vaccination. Diets were offered twice daily as total mixed rations, and animals had ad libitum access to feed and automatic drinking water. No additional diets or feed additives outside the experimental treatments were provided during the trial. The experimental diets were formulated on a dry matter basis according to the Nutrient Requirements of Beef cattle (NRC, 2016) and were designed to be isonitrogenous and isoenergetic. The ingredient composition, nutrient composition, and carbohydrate composition of the diets are presented in Table 1, Table 2 and Table 3, respectively.

### 2.2. Growth Performance and Feed Efficiency

Animals were weighed after fasting on day 1 and at the end of each finishing duration: day 300 for T10, day 360 for T12, and day 420 for T14. Average daily gain was calculated as the difference between final and initial body weight divided by the number of feeding days. Feed offered and refusals were recorded for three consecutive days at the beginning of the trial and immediately before each finishing endpoint. Average dry matter intake was calculated as the difference between dry matter offered and refusals. Feed-to-gain ratio was calculated as dry matter intake divided by average daily gain.

Production cost per kilogram of meat was calculated according to feed cost, body weight gain, and carcass/meat production data. Cost per kg of body weight gain = Total feed cost (RMB) / Total weight gain (kg).

### 2.3. Apparent Total Tract Digestibility Measurements

Fecal samples were collected for three consecutive days before the end of each finishing period. Fresh feces were collected before morning feeding, at 12:00, and before evening feeding. Approximately 500 g of feces was collected at each sampling time, treated with 10% sulfuric acid for nitrogen fixation, and stored at −20 °C. Samples collected over three days were pooled for each animal, mixed thoroughly, subsampled, dried at 65 ± 5 °C, ground, and stored for analysis [20].

Dry matter (DM), crude protein (CP), ether extract (EE), neutral detergent fiber (NDF), and acid detergent fiber (ADF) were determined using standard feed analysis procedures. Acid-insoluble ash (AIA) was used as an internal marker to calculate apparent nutrient digestibility according to the following formula:

Apparent nutrient digestibility (%) = 100 − [(marker concentration in TMR/marker concentration in feces) × (nutrient concentration in feces/nutrient concentration in TMR) × 100]).

### 2.4. Slaughter Performance and Meat Quality

At each finishing endpoint, animals were slaughtered according to commercial slaughter procedures. Pre-slaughter weight, carcass weight, lean meat weight, eye muscle area, and backfat thickness were measured. Dressing percentage and lean meat yield were calculated. Meat quality traits were measured according to previously described procedures with minor modifications [21].

Meat quality traits were measured using samples of the longissimus thoracis muscle collected between the 12th and 13th ribs from each carcass. Carcasses were chilled at 0–4 °C for 48 h before meat sampling. Meat color was evaluated on the freshly cut surface of the longissimus thoracis after exposure to air for 30 min to allow blooming. Color parameters, including lightness (L*), redness (a*), and yellowness (b*), were measured using a portable colorimeter (CR-400, Konica Minolta Sensing Inc., Osaka, Japan) calibrated with a standard white tile before measurement. Three readings were taken at different positions on each sample, and the average value was used for statistical analysis.

Muscle pH was measured at 45 min and 24 h postmortem using a portable pH meter (HI99163, Hanna Instruments, Woonsocket, RI, USA) equipped with a penetrating electrode. The pH meter was calibrated with standard buffer solutions at pH 4.00 and 7.00 before measurement. The electrode was inserted into the center of the longissimus thoracis muscle, and three readings were taken for each sample. The 24 h pH was considered the ultimate pH.

Cooking loss was measured as a water-holding capacity-related trait. Briefly, meat samples were weighed before cooking, cooked until the internal temperature reached 70 °C, cooled to room temperature, and reweighed. Cooking loss was calculated as follows: cooking loss (%) = [(raw weight − cooked weight)/raw weight] × 100. Warner–Bratzler shear force was measured using a texture analyzer equipped with a Warner–Bratzler shear blade (TA.XTplus, Stable Micro Systems Ltd., Godalming, UK). Three cores were taken parallel to the muscle fiber direction from each cooked sample, and the average value was used for analysis.

### 2.5. Blood Biochemical Analysis

Before the end of each finishing period, blood samples were collected from the jugular vein of fasted animals using 10 mL vacuum tubes. Samples were allowed to stand until serum separation, centrifuged at 3500 r/min for 15 min, and the serum was stored at −20 °C until analysis. Serum total protein, albumin, glucose, triglyceride, urea, alanine aminotransferase, aspartate aminotransferase, and alkaline phosphatase were measured using an automatic biochemical analyzer (Hitachi 3100, Hitachi High-Technologies Corporation, Tokyo, Japan) with commercial assay kits according to the manufacturer’s instructions [22].

### 2.6. Rumen Fermentation Parameters

Rumen fluid was collected immediately after slaughter. Samples were filtered through four layers of sterile gauze. Fresh rumen fluid pH was measured immediately using a portable pH meter. Subsamples were stored at −20 °C for ammonia nitrogen and volatile fatty acid analysis.

Ammonia nitrogen was determined using a spectrophotometric method [23]. For volatile fatty acid analysis, 1.5 mL of thawed rumen fluid was centrifuged at 10,000 r/min at 4 °C for 10 min. Then, 1 mL of supernatant was mixed with 0.2 mL of 25% metaphosphoric acid, allowed to stand for 1 h, and centrifuged again. The supernatant was analyzed using gas chromatography with a flame ionization detector and a DB-FFAP column. The oven temperature started at 65 °C and increased to 190 °C at 20 °C/min. The split ratio was 50:1, and the injection volume was 1 μL. Acetate, propionate, butyrate, isobutyrate, valerate, isovalerate, total volatile fatty acids, and the acetate-to-propionate ratio were calculated [24].

### 2.7. Rumen Microbiota Analysis

Rumen fluid samples were collected immediately after slaughter, filtered through four layers of sterile gauze, transferred into 50 mL cryotubes, rapidly frozen in liquid nitrogen, and stored at −80 °C. Microbial DNA was extracted from rumen fluid samples. The V3–V4 region of the bacterial 16S rRNA gene was amplified using the forward primer ACTCCTACGGGAGGCAGCAG and reverse primer GGACTACHVGGGTWTCTAAT [25]. PCR amplification was performed under the following conditions: initial denaturation at 98 °C for 30 s; 32 cycles of 98 °C for 10 s, 54 °C for 30 s, and 72 °C for 45 s; and final extension at 72 °C for 10 min.

PCR products were purified using AMPure XP beads, quantified using Qubit, and subjected to library quality control. Sequencing was performed using the Illumina MiSeq platform. Raw reads were processed using the QIIME2 pipeline [26]. Primer sequences were removed using cutadapt [27]; paired-end reads were merged using vsearch; low-quality sequences and chimeric sequences were removed using vsearch and the RDP chimera database. Alpha diversity was evaluated using Shannon, Simpson, Chao1, and observed species indices. Beta diversity was evaluated using Bray–Curtis distance and principal coordinate analysis. Linear discriminant analysis effect size was used to identify differential taxa, with an LDA threshold of 3 [28]. Functional prediction was performed using Tax4Fun2 and KEGG annotation [29]. Spearman correlation analysis was conducted between the top 20 rumen bacterial genera and rumen fermentation parameters.

### 2.8. Statistical Analysis

Data were checked for normality and homogeneity of variance before analysis. Growth performance, nutrient digestibility, carcass traits, meat quality, blood biochemical parameters, and rumen fermentation data were analyzed using two-way analysis of variance with feeding regime, finishing duration, and their interaction as fixed effects. Tukey’s multiple comparison test was used for post hoc comparisons. Results are presented as means ± standard deviation or means with pooled standard error of the mean, as appropriate. Differences were considered significant at *p* < 0.05, and tendencies were discussed at 0.05 ≤ *p* < 0.10 where relevant.

For rumen microbiota, alpha diversity was compared among groups using appropriate non-parametric or parametric tests according to data distribution. Beta diversity was evaluated based on Bray–Curtis distance and visualized by principal coordinate analysis. Associations between bacterial genera and rumen fermentation variables were assessed using Spearman correlation analysis. In the final statistical analysis for publication, pen should be carefully considered as the experimental unit for feed intake and feed efficiency when these variables are recorded at the pen level.

## 3. Results

### 3.1. Growth Performance, Feed Efficiency, and Production Cost

Feeding regime and finishing duration jointly affected growth performance in Black Angus bulls. As shown in Table 4, a significant feeding regime × finishing duration interaction was observed for average daily gain, final body weight, and feed-to-gain ratio. At T10, bulls fed the CSRC total mixed ration (TMR) had greater average daily gain than those fed the RSC TMR (1.54 ± 0.03 vs. 1.49 ± 0.02 kg/d). At T12, average daily gain was similar between CSRC and RSC bulls (1.39 ± 0.03 vs. 1.40 ± 0.02 kg/d). At T14, average daily gain declined in both groups, with values of 1.28 ± 0.03 and 1.31 ± 0.02 kg/d for CSRC and RSC bulls, respectively.

Final body weight increased with finishing duration (Table 4). At T10, final body weight was 703.25 ± 8.65 kg in the CSRC group and 695.89 ± 10.63 kg in the RSC group. At T12, final body weight was 736.37 ± 22.80 kg in the CSRC group and 758.52 ± 15.32 kg in the RSC group. At T14, RSC bulls reached a higher final body weight than CSRC bulls (805.71 ± 15.24 vs. 780.99 ± 16.88 kg).

Dry matter intake did not differ significantly among treatment combinations and ranged from 10.36 to 10.79 kg/d. However, feed-to-gain ratio increased with finishing duration (Table 4). At T10, CSRC bulls had a lower feed-to-gain ratio than RSC bulls (6.73 ± 0.18 vs. 7.15 ± 0.25), indicating better feed efficiency during early finishing. At T14, feed-to-gain ratio was high in both groups, reaching 8.30 ± 0.34 in CSRC bulls and 8.21 ± 0.26 in RSC bulls.

Production cost per kilogram of meat increased with finishing duration in both feeding regimes (Table 5). In the CSRC group, production cost increased from 11.73 ± 0.31 yuan/kg at T10 to 13.25 ± 0.36 yuan/kg at T12 and 14.42 ± 0.59 yuan/kg at T14. In the RSC group, production cost increased from 12.22 ± 0.42 yuan/kg at T10 to 13.09 ± 0.41 yuan/kg at T12 and 13.96 ± 0.44 yuan/kg at T14. These results indicate that the CSRC TMR had a cost advantage during early finishing, whereas the RSC TMR became more favorable during late finishing.

### 3.2. Apparent Total Tract Digestibility of Nutrients

As shown in Table 6, feeding regime alone did not significantly affect the apparent digestibility of dry matter, crude protein, ether extract, neutral detergent fiber, or acid detergent fiber. In contrast, finishing duration significantly reduced nutrient digestibility. Dry matter digestibility declined from 75.87 ± 1.87% at T10 to 70.54 ± 2.85% at T14 in the CSRC group, and from 76.52 ± 2.43% at T10 to 72.18 ± 2.97% at T14 in the RSC group.

Crude protein digestibility decreased with finishing duration in both groups. In the CSRC group, crude protein digestibility decreased from 79.47 ± 1.60% at T10 to 69.10 ± 1.52% at T14. In the RSC group, it decreased from 77.28 ± 2.04% at T10 to 70.51 ± 1.73% at T14. Ether extract digestibility also decreased at late finishing, especially in the RSC group, where it declined from 80.18 ± 2.78% at T10 to 71.82 ± 1.81% at T14.

Fiber digestibility showed a pronounced decline as finishing duration increased. Neutral detergent fiber digestibility decreased from 70.14 ± 2.06% to 64.53 ± 2.00% in the CSRC group and from 72.28 ± 1.45% to 61.91 ± 1.87% in the RSC group. Acid detergent fiber digestibility decreased from 40.22 ± 0.92% to 34.90 ± 1.19% in the CSRC group and from 41.33 ± 1.51% to 32.91 ± 0.61% in the RSC group. Significant feeding regime × finishing duration interactions were observed for ether extract, neutral detergent fiber, and acid detergent fiber digestibility (Table 6).

### 3.3. Slaughter Performance and Meat Quality

Table 7 shows the effects of feeding regime and finishing duration on carcass traits in Black Angus bulls. Finishing duration significantly improved several carcass traits. Pre-slaughter weight increased from 654.20 ± 29.16 kg at T10 to 752.38 ± 34.69 kg at T14 in the CSRC group and from 661.80 ± 32.82 kg to 758.63 ± 49.28 kg in the RSC group. Carcass weight increased from 376.79 ± 26.00 kg at T10 to 408.60 ± 22.96 kg at T14 in the CSRC group and from 375.99 ± 13.43 kg to 433.48 ± 21.34 kg in the RSC group. The RSC group had greater overall carcass weight than the CSRC group.

**Table 7:** Interactive effects of feeding regime and finishing duration on carcass traits in Black Angus bulls.

Items	Feeding Regime		Finishing Duration			SEM	Significance		
	CSRC	RSC	T10	T12	T14		FR	FD	FR × FD
Pre-slaughter weight, kg	701.61	714.84	658 ^c^	711.17 ^b^	755.51 ^a^	6.78	NS	***	NS
Torso weight, kg	397.11 ^b^	416.68 ^a^	376.39 ^b^	423.26 ^a^	421.04 ^a^	4.46	**	***	*
Slaughter rate, %	56.76	58.40	57.31 ^ab^	59.56 ^a^	55.89 ^b^	0.56	NS	*	NS
Lean meat yield, %	40.80	41.38	43.21 ^a^	40.55 ^b^	39.51 ^b^	0.35	NS	***	NS
Eye muscle area, cm^2^	105.86 ^b^	111.00 ^a^	105.35 ^b^	107.44 ^b^	112.51 ^a^	0.7	***	***	*
Backfat thickness, cm	1.17	1.20	1.01 ^c^	1.23 ^b^	1.31 ^a^	0.02	NS	***	NS

Note: CSRC, corn silage–rice straw–concentrate TMR; RSC, rice straw–concentrate TMR. T10, finishing durations were 10 months; T12, finishing durations were 12 months; T14, finishing durations were 14; FR, feeding regime; FD, finishing duration; FR × FD, interaction between feeding regime and finishing duration. Values within the same row with different superscript letters differ significantly, whereas values with the same superscript letters do not differ significantly. *, **, and *** indicate significant differences at *p* < 0.05, *p* < 0.01, and *p* < 0.001, respectively; NS indicates no significant difference.

Dressing percentage showed a non-linear response to finishing duration. In the RSC group, dressing percentage was highest at T12 (60.98 ± 3.89%). Lean meat yield decreased with finishing duration. In the CSRC group, lean meat yield decreased from 43.22 ± 2.19% at T10 to 38.82 ± 1.79% at T14. In the RSC group, it decreased from 43.20 ± 3.39% to 40.19 ± 1.99%.

Eye muscle area increased with finishing duration and was generally higher in the RSC group. At T14, eye muscle area reached 110.86 ± 4.98 cm^2^ in the CSRC group and 114.16 ± 2.52 cm^2^ in the RSC group. Backfat thickness also increased with finishing duration, from approximately 1.01 cm at T10 to 1.27 ± 0.10 cm in the CSRC group and 1.35 ± 0.04 cm in the RSC group at T14.

Meat color improved with finishing duration (Table 8). Lightness increased from approximately 27.5 at T10 to more than 30.9 at T14 in both groups. Redness increased markedly with finishing duration and was highest in the RSC group at T14, reaching 12.01 ± 0.48. Yellowness also increased slightly with finishing duration, with the highest value observed in the RSC group at T14.

**Table 8:** Interactive effects of feeding regime and finishing duration on meat quality traits in Black Angus bulls.

Items	Feeding Regime		Finishing Duration			SEM	Significance		
	CSRC	RSC	T10	T12	T14		FR	FD	FR × FD
Lightness (L*)	30.00 ^a^	29.24 ^b^	27.61 ^c^	29.86 ^b^	31.40 ^a^	0.27	*	***	NS
Redness (a*)	8.92 ^b^	9.85 ^a^	8.11 ^c^	9.05 ^b^	11.00 ^a^	0.18	***	***	***
Yellowness (b*)	1.30	1.31	1.26 ^b^	1.29 ^b^	1.36 ^a^	0.01	NS	**	NS
Cooking loss (%)	42.25 ^a^	40.12 ^b^	42.10	40.84	40.61	0.33	***	NS	*
Shear force (kgf)	1.60	1.58	1.62 ^a^	1.60 ^ab^	1.55 ^b^	0.01	NS	**	NS
pH at 45 min	6.32	6.41	6.47	6.32	6.30	0.03	NS	NS	NS

Note: CSRC, corn silage–rice straw–concentrate TMR; RSC, rice straw–concentrate TMR. T10, finishing durations were 10 months; T12, finishing durations were 12 months; T14, finishing durations were 14; FR, feeding regime; FD, finishing duration; FR × FD, interaction between feeding regime and finishing duration. Values within the same row with different superscript letters differ significantly, whereas values with the same superscript letters do not differ significantly. *, **, and *** indicate significant differences at *p* < 0.05, *p* < 0.01, and *p* < 0.001, respectively; NS indicates no significant difference.

Cooking loss was affected by feeding regime and its interaction with finishing duration. At T14, cooking loss was lower in the RSC group than in the CSRC group (38.65 ± 2.29% vs. 42.57 ± 2.16%), suggesting better water-holding capacity in the late-finished RSC bulls. Shear force decreased with finishing duration, indicating improved tenderness. In the CSRC group, shear force decreased from 1.64 ± 0.10 kgf at T10 to 1.53 ± 0.07 kgf at T14. In the RSC group, shear force decreased from 1.61 ± 0.07 kgf to 1.56 ± 0.07 kgf. Meat pH at 45 min and 24 h was not significantly affected by feeding regime or finishing duration (Table 8).

### 3.4. Blood Biochemical Parameters

As shown in Table 9, serum biochemical parameters reflected changes in protein, energy, lipid, nitrogen metabolism, and liver-related indicators during finishing. Serum total protein decreased with finishing duration in both groups. In the CSRC group, total protein decreased from 96.03 ± 2.75 g/L at T10 to 67.13 ± 3.79 g/L at T14. In the RSC group, it decreased from 96.57 ± 5.53 g/L to 70.04 ± 2.28 g/L. Serum albumin showed a similar decreasing trend, and overall albumin was lower in the CSRC group than in the RSC group.

Serum alanine aminotransferase, aspartate aminotransferase, and alkaline phosphatase increased with finishing duration. At T14, alanine aminotransferase reached 45.21 ± 2.10 U/L in the CSRC group and 41.49 ± 2.53 U/L in the RSC group. Aspartate aminotransferase reached 168.80 ± 5.56 U/L and 150.68 ± 7.82 U/L, respectively. Alkaline phosphatase reached 243.05 ± 12.42 U/L and 215.69 ± 8.60 U/L, respectively. These results suggest that prolonged finishing altered systemic metabolic status and increased liver-related enzyme activities.

Serum glucose increased with finishing duration in both groups. At T14, glucose concentration was higher in the CSRC group than in the RSC group (3.50 ± 0.43 vs. 3.20 ± 0.14 mmol/L). Serum triglyceride increased with finishing duration, and the RSC group showed the highest triglyceride concentration at T14. Serum urea also increased with finishing duration and was higher in the CSRC group than in the RSC group at corresponding stages, indicating differences in nitrogen metabolism between feeding regimes.

### 3.5. Rumen Fermentation Parameters

Rumen fermentation was markedly affected by both feeding regime and finishing duration (Table 10). Rumen pH decreased with finishing duration in both groups. In the CSRC group, pH decreased from 6.78 ± 0.26 at T10 to 5.73 ± 0.16 at T14. In the RSC group, pH decreased from 6.63 ± 0.19 to 6.05 ± 0.18. At T14, rumen pH was lower in the CSRC group than in the RSC group.

Ammonia nitrogen increased with finishing duration. In the CSRC group, NH_3_-N increased from 11.94 ± 0.40 mg/dL at T10 to 16.93 ± 0.63 mg/dL at T14. In the RSC group, it increased from 11.89 ± 0.26 mg/dL to 15.30 ± 0.30 mg/dL. At T12 and T14, NH_3_-N was higher in the CSRC group than in the RSC group.

The CSRC TMR promoted an acetate-oriented fermentation pattern. Across all finishing durations, acetate concentration was higher in the CSRC group than in the RSC group. Acetate decreased from 54.93 ± 1.91 mmol/L at T10 to 45.82 ± 1.19 mmol/L at T14 in the CSRC group, and from 50.04 ± 1.30 mmol/L to 41.39 ± 1.45 mmol/L in the RSC group. In contrast, propionate increased with finishing duration. At T14, propionate was markedly higher in the RSC group than in the CSRC group (23.89 ± 0.58 vs. 17.82 ± 0.62 mmol/L).

Butyrate, isobutyrate, valerate, and isovalerate generally decreased with finishing duration. Butyrate was consistently higher in the CSRC group than in the RSC group. Total volatile fatty acids were also higher in the CSRC group at T10 and T12. At T10, total volatile fatty acids were 78.41 ± 1.94 mmol/L in the CSRC group and 71.67 ± 1.72 mmol/L in the RSC group. At T12, they were 74.78 ± 1.36 mmol/L and 68.54 ± 1.37 mmol/L, respectively. At T14, total volatile fatty acids were similar between groups. The acetate-to-propionate ratio declined with finishing duration in both groups and remained higher in the CSRC group than in the RSC group, indicating that CSRC maintained a more acetate-oriented fermentation pattern, whereas RSC shifted more strongly toward propionate production during late finishing.

### 3.6. Rumen Microbial Diversity and Community Structure

**Figure 1:**
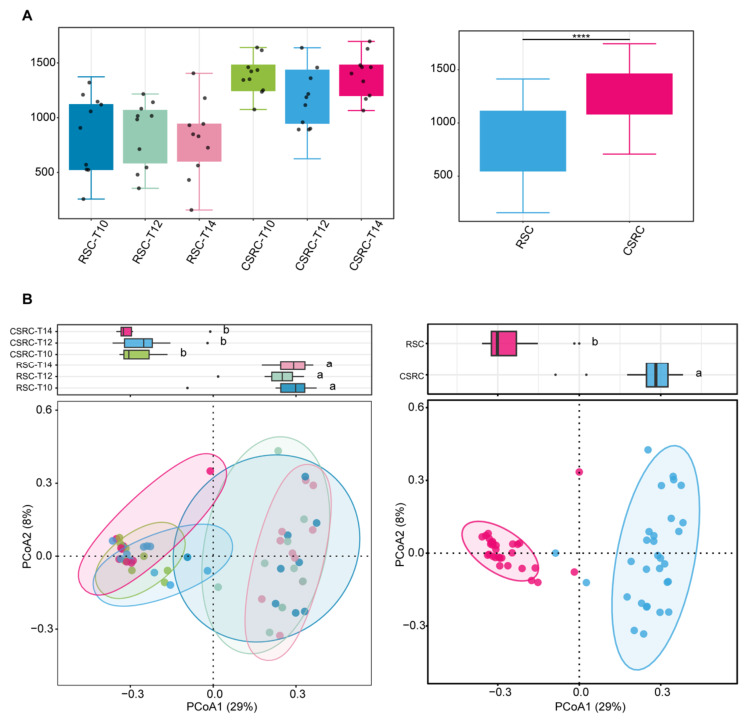
Effects of feeding regime and finishing duration on rumen microbial richness and community structure in Black Angus bulls. (**A**) Chao1 richness index of rumen microbiota among the six treatment combinations and between the two feeding regimes. (**B**) Principal coordinate analysis based on Bray–Curtis distance showing rumen bacterial community structure among the six treatment combinations and between the two feeding regimes. Ellipses represent the distribution of samples within each treatment group. CSRC, corn silage–rice straw–concentrate TMR; RSC, rice straw–concentrate TMR; T10, 10-month finishing duration; T12, 12-month finishing duration; T14, 14-month finishing duration. **** *p* < 0.0001. Different letters indicate significant differences among groups (*p* < 0.05).

Microbial richness analysis showed that finishing duration did not significantly alter rumen microbial diversity within the same feeding regime. However, the Chao1 richness index was higher in the CSRC group than in the RSC group, indicating that the inclusion of corn silage was associated with greater rumen microbial richness and community complexity (Figure 1A).

Principal coordinate analysis based on Bray–Curtis distance revealed a clear separation between the CSRC and RSC groups. In contrast, within the same feeding regime, rumen microbial community structure did not differ significantly among the different finishing durations (Figure 1B). These results suggest that feeding regime was the major factor shaping rumen bacterial community structure, while finishing duration exerted a weaker effect on beta diversity.

**Figure 2:**
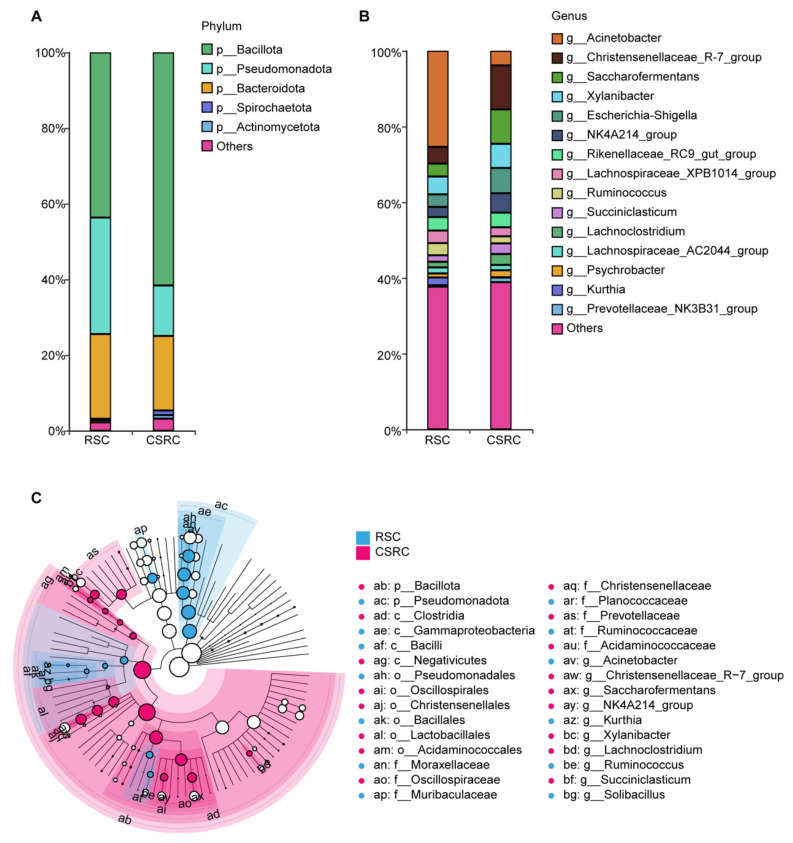
Effects of feeding regime on rumen bacterial composition and differential taxa in Black Angus bulls. (**A**) Relative abundance of rumen bacteria at the phylum level in the RSC and CSRC groups. (**B**) Relative abundance of the dominant rumen bacterial genera in the RSC and CSRC groups. (**C**) LEfSe cladogram showing bacterial taxa differentially enriched between the two feeding regimes. Colored nodes indicate taxa significantly enriched in the corresponding group, whereas white nodes indicate taxa with no significant difference. CSRC, corn silage–rice straw–concentrate TMR; RSC, rice straw–concentrate TMR. LEfSe, linear discriminant analysis effect size. Different letters indicate significant differences among groups (*p* < 0.05).

At the phylum level (Figure 2A), more than 95% of the bacterial sequences were mainly assigned to *Bacillota*, *Pseudomonadota*, *Bacteroidota*, *Spirochaetota*, and *Actinomycetota*. Compared with the RSC group, the CSRC group had significantly higher relative abundances of *Bacillota*, *Spirochaetota*, and *Actinomycetota*, but a significantly lower relative abundance of *Pseudomonadota*. No significant effect of finishing duration was observed on the overall phylum-level composition.

At the genus level (Figure 2B), among the five most abundant genera, the relative abundances of *Christensenellaceae_R-7_group*, *Saccharofermentans*, *NK4A214_group*, and *Kurthia* were significantly higher in the CSRC group than in the RSC group, whereas *Acinetobacter* was significantly lower in the CSRC group. LEfSe analysis further identified feeding regime-associated microbial biomarkers (Figure 2C), including *Saccharofermentans*, *NK4A214_group*, and *Acinetobacter*, indicating that the two dietary regimes selectively enriched distinct rumen bacterial taxa.

**Figure 3:**
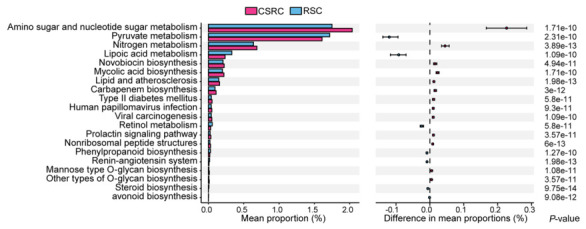
Predicted functional differences in rumen microbiota between the CSRC and RSC groups. Amino sugar and nucleotide sugar metabolism was the most significantly enriched pathway in the CSRC group. CSRC, corn silage–rice straw–concentrate TMR; RSC, rice straw–concentrate TMR. Functional profiles were predicted from 16S rRNA sequencing data.

Predicted functional profiling showed that amino sugar and nucleotide sugar metabolism was the most significantly enriched pathway among the differentially abundant pathways between groups, with higher enrichment in the CSRC group than in the RSC group (Figure 3). Because these functions were inferred from 16S rRNA sequencing data, this result should be interpreted as a prediction of microbial functional potential rather than direct functional evidence.

## 4. Discussion

Feeding regime and finishing duration are closely linked management factors that influence beef cattle performance through nutrient supply, rumen fermentation, and carcass development. The following discussion therefore focuses on the biological interpretation and practical implications of these combined effects.

The early advantage of the CSRC TMR may be attributed to the nutritional and fermentative characteristics of whole-plant corn silage. Compared with rice straw, whole-plant corn silage provides higher levels of fermentable carbohydrates, digestible fiber, and organic acids [6], thereby increasing the availability of substrates for rumen microorganisms and promoting microbial fermentation [7]. From a biochemical perspective, the greater supply of fermentable fiber and soluble carbohydrates can enhance the production of volatile fatty acids (VFAs), which are the main energy source for ruminants [30]. In particular, acetate and butyrate are closely related to fiber fermentation, epithelial energy metabolism, and lipid synthesis [31], whereas an adequate VFA supply supports energy availability for growth. This may explain the higher average daily gain and lower feed-to-gain ratio observed in CSRC-fed bulls during early finishing. In contrast, the greater final body weight of RSC-fed bulls at 14 months may be partly associated with the increased concentrate proportion during late finishing and the accompanying shift toward propionate-oriented rumen fermentation. Higher concentrate-to-forage ratios have been shown to promote ruminal propionate production [18,32], and because propionate is the major glucogenic precursor in ruminants, this fermentation pattern may have contributed to a greater glucogenic energy supply and subsequent body weight gain [33,34]. Rice hay, although lower in nutritive value than whole-plant corn silage, provides physically effective fiber that helps maintain rumen stability and supports efficient utilization of high-concentrate diets, thereby contributing to growth performance and carcass development [35,36]. This stable rumen environment and enhanced propionate production may also support glucogenic energy supply and fat deposition, which could partly explain the improved meat redness observed in RSC-fed bulls. In this context, rice straw should not be interpreted as a high-energy ingredient by itself. Rather, its role in the RSC TMR was likely to provide physically effective fiber, promote chewing and rumination, contribute to saliva buffering, and help maintain rumen fermentation stability in a concentrate-based finishing system. Thus, the favorable late-finishing performance of RSC-fed bulls was probably due to the complementary effects of rice straw as a structural roughage source and concentrate as an energy source. The decline in average daily gain and increase in feed-to-gain ratio with prolonged finishing are consistent with previous findings that growth efficiency decreases as bulls become older and heavier, because a greater proportion of nutrient intake is partitioned toward maintenance and fat accretion rather than lean tissue deposition [4,13]. At the same time, the composition of gain gradually shifts from lean tissue accretion toward fat deposition, which requires more energy per unit of tissue gain than protein deposition. Therefore, even when final body weight and carcass fatness increase, growth efficiency may decline. The reduced apparent total-tract digestibility of dry matter, crude protein, ether extract, neutral detergent fiber, and acid detergent fiber further suggests that late finishing was accompanied by lower nutrient utilization efficiency, especially reduced fiber digestion.

Although prolonged finishing reduced feed efficiency, it improved several carcass and meat quality traits through changes in tissue growth and energy partitioning. Increased carcass weight, eye muscle area, and backfat thickness indicate greater carcass development and lipid deposition. Physiologically, longer finishing allows more time for muscle growth, adipocyte development, and subcutaneous and intramuscular fat accumulation [4], although intramuscular fat was not directly measured in this study. These findings are consistent with previous studies showing that extended days on feed can increase hot carcass weight, carcass fatness, and the likelihood of achieving higher quality grades, although often at the expense of growth efficiency [4,13]. Greater fat deposition may contribute to improved meat quality by enhancing carcass insulation during chilling, reducing the risk of cold shortening, and improving tenderness-related traits. The increase in meat redness may be associated with greater muscle maturity and pigment concentration, whereas the decrease in shear force suggests improved tenderness, potentially related to changes in muscle structure, fat deposition, and postmortem proteolysis. However, prolonged finishing also reduced lean meat yield and increased production cost per kilogram of meat in this study. Therefore, extending the finishing period involves a biological and economic trade-off: it may improve carcass development and selected meat quality attributes, but it also reduces nutrient-use efficiency and increases production input [37,38].

The rumen fermentation and microbial results provide further mechanistic insight into the different responses to feeding regime. The CSRC TMR increased acetate, butyrate, total volatile fatty acid concentration, and the acetate-to-propionate ratio, indicating a more acetate-oriented fermentation pattern. This was consistent with the greater supply of structural and fermentable carbohydrates from whole-plant corn silage, which has been shown to improve rumen fermentation and microbial metabolism in bulls [7,8,39]. In contrast, prolonged finishing, especially under the RSC TMR, shifted rumen fermentation toward propionate production, likely because of the increased concentrate proportion and greater starch fermentation during late finishing [18]. Rumen microbiota analysis further showed that feeding regime was a stronger driver of bacterial community structure than finishing duration. CSRC-fed bulls had greater microbial richness and distinct bacterial composition, with higher abundances of several TMR-responsive taxa, including Christensenellaceae_R-7_group, *Saccharofermentans*, NK4A214_group, and *Kurthia*, and lower abundance of *Acinetobacter*. Among these taxa, Christensenellaceae_R-7_group and *Saccharofermentans* have been frequently associated with fiber degradation, carbohydrate fermentation, and rumen short-chain fatty acid-related processes [40,41,42,43]. Therefore, their enrichment may partly explain the higher VFA concentration and acetate-oriented fermentation observed in CSRC-fed bulls. It should be noted that the feed ingredients themselves were not evaluated for specific microbial populations before inclusion in the diets. Thus, the rumen microbiota results should be interpreted as dietary substrate-driven changes in the rumen microbial community rather than direct evidence of microbial transfer from feed ingredients. Functional prediction showed that amino sugar and nucleotide sugar metabolism was enriched in the CSRC group, suggesting greater microbial potential for carbohydrate utilization, cell-wall biosynthesis, and microbial biomass turnover. However, because these functions were inferred from 16S rRNA sequencing, they should be interpreted as predicted functional potential rather than direct evidence of microbial activity or causality [44].

These findings have practical implications for Black Angus finishing systems using local roughage resources. The CSRC TMR appears more suitable for early finishing, when improving daily gain, feed conversion, and production cost is the primary goal. In contrast, the RSC TMR may be more advantageous during late finishing, when final body weight, meat redness, and cost control become more important. Under the present experimental conditions, a 12-month finishing duration may represent a practical compromise between production efficiency and meat quality. Nevertheless, several limitations should be noted. First, the trial was conducted under commercial farm conditions, and pen-level or housing effects may have influenced some performance variables. Second, if feed intake was recorded at the pen level, pen should be considered the experimental unit for dry matter intake and feed efficiency. Third, microbial function was inferred from 16S rRNA sequencing and requires validation using metagenomics, metatranscriptomics, or metabolomics. Finally, intramuscular fat content, fatty acid composition, flavor compounds, sensory evaluation, methane emissions, and life-cycle environmental impacts were not measured. Future studies integrating animal performance, meat quality, rumen multi-omics, economic analysis, and environmental assessment will help develop more precise and sustainable finishing strategies for Black Angus bulls.

## 5. Conclusions

The results of this study support a stage-specific feeding strategy for Black Angus bull finishing. Under the present experimental conditions, the corn silage-rice straw-concentrate total mixed ration (TMR) is more suitable for early finishing when improving growth efficiency is the main objective, whereas the rice straw–concentrate TMR may be more appropriate during late finishing when final body weight, meat quality, and feed cost are considered together. Extending the finishing duration should be carefully balanced against reduced feed efficiency and increased production input; therefore, a 12-month finishing duration may represent a practical compromise for achieving acceptable production efficiency, meat quality, and economic return.

## Figures and Tables

**Table 1:** Dietary ingredient composition of experimental diets under different feeding regimes and finishing durations.

Items	CSRC			RSC		
	T10	T12	T14	T10	T12	T14
Corn silage	20.00	25.00	30.00	-	-	-
Rice hay	15.60	8.60	1.60	26.20	21.93	17.66
Corn germ meal	13.00	13.00	13.00	16.00	16.00	16.00
Corn gluten feed, dry	11.00	12.50	14.00	11.7	14.12	16.53
Corn gluten meal	7.40	4.70	2.00	6.00	3.00	-
Corn grain	29.20	32.50	35.8	36.1	41.03	45.96
Calcium carbonate	2.00	1.70	1.40	2.00	1.70	1.40
Salt	0.40	0.40	0.40	0.40	0.40	0.40
Sodium bicarbonate	0.60	0.80	1.00	0.60	0.80	1.00
Slow-release urea (200)	0.40	0.40	0.40	0.60	0.62	0.65
Premix ^1^	0.40	0.40	0.40	0.40	0.40	0.40
Total	100.00	100.00	100	100	100	100

^1^ Values are expressed on a dry matter basis unless otherwise stated. CSRC, corn silage–rice straw–concentrate TMR; RSC, rice straw–concentrate TMR; T10, finishing durations were 10 months; T12, finishing durations were 12 months; T14, finishing durations were 14 months. Each kilogram of premix contained Co, 41.25 mg; Cu, 2750 mg; I, 137.5 mg; Fe, 13,750 mg; Mn, 5500 mg; Se, 27.5 mg; Zn, 8250 mg; vitamin A, 1,155,000 IU; vitamin D3, 78,375 IU; and vitamin E, 10,037.5 IU.

**Table 2:** Nutrient composition of experimental diets (%DM).

Items	CSRC			RSC		
	250–400 kg	400–550 kg	550 kg to Slaughter	250–400 kg	400–550 kg	550 kg to Slaughter
Crude Protein	16.59	15.27	13.95	16.60	15.26	13.93
RUP	8.36	7.29	6.23	8.34	7.28	6.22
MP	11.09	9.55	9.22	11.05	9.75	9.28
TDN	73.16	74.43	75.71	73.15	74.43	75.71
NDF	30.73	29.15	27.56	30.73	29.15	27.57
PeNDF	20.79	18.46	16.13	20.97	18.73	16.48
Ca	0.99	0.87	0.75	0.97	0.85	0.72
P	0.53	0.54	0.55	0.56	0.57	0.59
Ether Extract	4.08	4.23	4.38	4.22	4.34	4.46
ME (Mcal/kg)	2.64	2.69	2.74	2.64	2.69	2.74

Note: CSRC, corn silage–rice straw–concentrate TMR; RSC, rice straw–concentrate TMR. RUP, Rumen Undegradable Protein; MP, Metabolizable Protein; TDN, Total Digestible Nutrients; NDF, Neutral Detergent Fiber; PeNDF, Physically Effective Neutral Detergent Fiber; ME, Metabolizable Energy.

**Table 3:** Dietary carbohydrate composition of experimental diets (%DM).

Items	CSRC			RSC		
	250–400 kg	400–550 kg	550 kg to Slaughter	250–400 kg	400–550 kg	550 kg to Slaughter
Carbohydrate	71.52	73.50	75.49	70.63	72.41	74.19
NDF	30.73	29.15	27.56	30.73	29.15	27.57
NFC	40.79	44.36	47.93	39.91	43.26	46.62
Pectin	4.58	4.62	4.67	4.51	4.61	4.69
Sugars	1.69	2.00	2.30	0.98	1.13	1.27
Starch	34.52	37.74	40.95	34.41	37.53	40.65
Lignin	2.26	2.16	2.06	2.17	2.04	1.91

Note: CSRC, corn silage–rice straw–concentrate TMR; RSC, rice straw–concentrate TMR. NDF, Neutral Detergent Fiber; NFC, Non-Fiber Carbohydrate.

**Table 4:** Interactive effects of feeding regime and finishing duration on growth performance in Black Angus bulls.

Growth Performance	Feeding Regime		Finishing Duration			SEM	Significance		
	CSRC	RSC	T10	T12	T14		FR	FD	FR × FD
Initial BW, Kg	239.63 ^b^	252.40 ^a^	245.04	245.40	247.60	3.26	***	NS	NS
Average daily gain, Kg	1.40	1.40	1.52 ^a^	1.39 ^b^	1.3 ^c^	0.007	NS	***	***
Final BW, Kg	740.20 ^b^	753.37 ^a^	699.57 ^c^	747.44 ^b^	793.35 ^a^	4.03	**	***	**
Dry Matter Intake, DMI	10.53	10.73	10.51	10.66	10.72	0.04	NS	NS	NS
F/G	7.56 ^b^	7.68 ^a^	6.94 ^c^	7.65 ^b^	8.25 ^a^	0.06	*	***	**

Note: CSRC, corn silage–rice straw–concentrate TMR; RSC, rice straw–concentrate TMR. T10, finishing durations were 10 months; T12, finishing durations were 12 months; T14, finishing durations were 14; FR, feeding regime; FD, finishing duration; FR × FD, interaction between feeding regime and finishing duration; BW, body weight; F/G, feed-to-gain ratio. Values within the same row with different superscript letters differ significantly, whereas values with the same superscript letters do not differ significantly. *, **, and *** indicate significant differences at *p* < 0.05, *p* < 0.01, and *p* < 0.001, respectively; NS indicates no significant difference.

**Table 5:** Production cost per kilogram of meat under different feeding regimes and finishing durations.

Items	CSRC			RSC		
	T10	T12	T14	T10	T12	T14
Production cost (yuan/kg)	11.73 ± 0.31 ^c^	13.25 ± 0.36 ^b^	14.42 ± 0.59 ^a^	12.22 ± 0.42 ^c^	13.09 ± 0.41 ^b^	13.96 ± 0.44 ^a^

Note: CSRC, corn silage–rice straw–concentrate TMR; RSC, rice straw–concentrate TMR. T10, finishing durations were 10 months; T12, finishing durations were 12 months; T14, finishing durations were 14. Values within the same row with different superscript letters differ significantly, whereas values with the same superscript letters do not differ significantly.

**Table 6:** Interactive effects of feeding regime and finishing duration on apparent total-tract nutrient digestibility in Black Angus bulls.

Items	Feeding Regime		Finishing Duration			SEM	Significance		
	CSRC	RSC	T10	T12	T14		FR	FD	FR × FD
Dry Matter	73.16	73.41	76.19 ^a^	72.31 ^b^	71.36 ^b^	0.59	NS	***	NS
Crude Protein	74.46	74.45	78.37 ^a^	75.18 ^b^	69.80 ^c^	0.48	NS	***	NS
Ether Extract	77.93	77.33	80.10 ^a^	79.25 ^a^	73.54 ^b^	0.52	NS	***	***
Neutral Detergent Fiber	67.54	67.74	71.21 ^a^	68.49 ^b^	63.22 ^c^	0.47	NS	***	***
Acid Detergent Fiber	37.68	36.81	40.67 ^a^	37.34 ^b^	33.90 ^c^	0.31	NS	***	***

Note: CSRC, corn silage–rice straw–concentrate TMR; RSC, rice straw–concentrate TMR. T10, finishing durations were 10 months; T12, finishing durations were 12 months; T14, finishing durations were 14; FR, feeding regime; FD, finishing duration; FR × FD, interaction between feeding regime and finishing duration. Values within the same row with different superscript letters differ significantly, whereas values with the same superscript letters do not differ significantly. *** indicate significant differences at *p* < 0.001; NS indicates no significant difference.

**Table 9:** Interactive effects of feeding regime and finishing duration on serum biochemical parameters in Black Angus bulls.

Items	Feeding Regime		Finishing Duration			SEM	Significance		
	CSRC	RSC	T10	T12	T14		FR	FD	FR × FD
Total protein, g/L	80.01	81.91	96.30 ^a^	78.00 ^b^	68.58 ^c^	1.58	NS	***	NS
Albumin, g/L	34.53 ^b^	35.7 ^a^	38.48 ^a^	35.42 ^b^	31.46 ^c^	0.45	*	***	**
Alanine aminotransferase, U/L	36.77	36.79	30.78 ^c^	36.21 ^b^	43.35 ^a^	0.73	NS	***	***
Aspartate aminotransferase, U/L	125.13 ^a^	115.87 ^b^	87.69 ^c^	114.07 ^b^	159.74 ^a^	4.00	***	***	***
Alkaline phosphatase, U/L	181.22 ^a^	170.93 ^b^	133.76 ^c^	165.10 ^b^	229.37 ^a^	5.38	***	***	***
Glucose, mmol/L	3.12 ^a^	2.92 ^b^	2.7 ^c^	3.00 ^b^	3.35 ^a^	0.05	**	***	NS
Triglyceride, mmol/L	0.127	0.126	0.08 ^c^	0.13 ^b^	0.17 ^a^	0.005	NS	***	***

Note: CSRC, corn silage–rice straw–concentrate TMR; RSC, rice straw–concentrate TMR. T10, finishing durations were 10 months; T12, finishing durations were 12 months; T14, finishing durations were 14; FR, feeding regime; FD, finishing duration; FR × FD, interaction between feeding regime and finishing duration. Values within the same row with different superscript letters differ significantly, whereas values with the same superscript letters do not differ significantly. *, **, and *** indicate significant differences at *p* < 0.05, *p* < 0.01, and *p* < 0.001, respectively; NS indicates no significant difference.

**Table 10:** Interactive effects of feeding regime and finishing duration on rumen fermentation parameters in Black Angus bulls.

Items	Feeding Regime		Finishing Duration			SEM	Significance		
	CSRC	RSC	T10	T12	T14		FR	FD	FR × FD
pH	6.24 ^b^	6.38 ^a^	6.70 ^a^	6.35 ^b^	5.89 ^c^	0.05	**	***	***
NH_3_-N (mg/dL)	14.33 ^a^	13.41 ^b^	11.91 ^c^	13.57 ^b^	16.12 ^a^	0.24	***	***	***
Acetate (mmol/L)	50.42 ^a^	45.42 ^b^	52.48 ^a^	47.67 ^b^	43.61 ^c^	0.60	***	***	NS
Propionate (mmol/L)	14.87 ^b^	17.32 ^a^	11.89 ^c^	15.53 ^b^	20.85 ^a^	0.54	***	***	***
Butyrate (mmol/L)	6.37 ^a^	5.01 ^b^	7.03 ^a^	5.56 ^b^	4.47 ^c^	0.17	***	***	***
Isobutyric acid (mmol/L)	0.99 ^a^	0.86 ^b^	1.14 ^a^	0.89 ^b^	0.75 ^c^	0.02	***	***	*
Valerate (mmol/L)	0.63 ^a^	0.57 ^b^	0.79 ^a^	0.55 ^b^	0.45 ^c^	0.02	***	***	***
Isovalerate (mmol/L)	1.54 ^a^	1.46 ^b^	1.71 ^a^	1.46 ^b^	1.34 ^c^	0.02	***	***	***
TVFA (mmol/L)	74.81 ^a^	70.64 ^b^	75.04 ^a^	71.66 ^b^	71.47 ^b^	0.45	***	***	***
Acetate/Propionate	3.54 ^a^	2.89 ^b^	4.42 ^a^	3.08 ^b^	2.15 ^c^	0.13	***	***	**

Note: CSRC, corn silage–rice straw–concentrate TMR; RSC, rice straw–concentrate TMR. T10, finishing durations were 10 months; T12, finishing durations were 12 months; T14, finishing durations were 14; FR, feeding regime; FD, finishing duration; FR × FD, interaction between feeding regime and finishing duration; TVFA, total volatile fatty acid. Values within the same row with different superscript letters differ significantly, whereas values with the same superscript letters do not differ significantly. *, **, and *** indicate significant differences at *p* < 0.05, *p* < 0.01, and *p* < 0.001, respectively; NS indicates no significant difference.

## Data Availability

The original contributions presented in this study are included in the article. Further inquiries can be directed to the corresponding author(s).

## References

[1] Drachmann F.F., Christensen M., Esberg J., Lauridsen T., Fogh A., Young J.F., Therkildsen M. (2024). Beef-on-dairy: Meat quality of veal and prediction of intramuscular fat using the Q-FOM™ Beef camera at the 5th–6th thoracic vertebra. Meat Sci..

[2] Liu J., Ellies-Oury M.-P., Stoyanchev T., Hocquette J.-F. (2022). Consumer perception of beef quality and how to control, improve and predict it? Focus on eating quality. Foods.

[3] Yang M., Ji K., Liu X., Wang Z., Lin X., Zhao H., Zhang X. (2025). Effects of resveratrol on growth performance, meat quality, and intramuscular fat deposition in finishing steers. J. Anim. Sci..

[4] Sperber J.L., Bondurant R.G., Erickson G.E., Bruns K., Funston R.N., MacDonald J.C. (2024). Effect of extended days on feed on carcass gain, efficiency, and quality of individually fed beef steers. Transl. Anim. Sci..

[5] Wan Y., Wang Y. (2025). Study on Comprehensive Utilization of Crop Straw and Spatial Distribution of Cattle and Sheep in China: 1978–2023. Agriculture.

[6] Zhang H., Zhang L., Xue X., Zhang X., Wang H., Gao T., Phillips C. (2021). Effect of feeding a diet comprised of various corn silages inclusion with peanut vine or wheat straw on performance, digestion, serum parameters and meat nutrients in finishing beef cattle. Anim. Biosci..

[7] Cui Y., Liu H., Gao Z., Xu J., Liu B., Guo M., Yang X., Niu J., Zhu X., Ma S. (2022). Whole-plant corn silage improves rumen fermentation and growth performance of beef cattle by altering rumen microbiota. Appl. Microbiol. Biotechnol..

[8] Ma J., Liu H., Liu M., Xu J., Lu J., Cao S., Li S., Ma S., Wang Z., Zhu X. (2023). Effects of diets combining peanut vine and whole-plant corn silage on growth performance, meat quality and rumen microbiota of simmental crossbred cattle. Foods.

[9] Aquino D., Del Barrio A., Trach N., Hai N., Khang D., Toan N., Gummert M., Hung N.V., Chivenge P., Douthwaite B. (2020). Rice straw-based fodder for ruminants. Sustainable Rice Straw Management.

[10] Kaeokliang O., Kawashima T., Narmseelee R., Butcha P., Sunato S., Thinowong A., Jindatajak Y. (2019). Effects of physically effective fiber in diets based on rice straw and cassava pulp on chewing activity, ruminal fermentation, milk production, and digestibility in dairy cows. Anim. Sci. J..

[11] Gao Q., Liu H., Wang Z., Lan X., An J., Shen W., Wan F. (2022). Recent advances in feed and nutrition of beef cattle in China—A review. Anim. Biosci..

[12] Galyean M., Nichols W., Streeter M., Hutcheson J. (2023). Effects of extended days on feed on rate of change in performance and carcass characteristics of feedlot steers and heifers and Holstein steers. Appl. Anim. Sci..

[13] Husz T., Word A., Karr K., Holland B., Lawrence T., Perkins T., Hutcheson J., Walter L. (2024). Effects of days on feed on performance and carcass characteristics of crossbred beef× dairy heifers. Appl. Anim. Sci..

[14] Ferreira I.M., Oliveira K.A., Cidrini I.A., de Abreu M.J.I., Sousa L.M., Batista L.H.C., Homem B.G.C., Prados L.F., Siqueira G.R., Resende F.D.d. (2023). Performance, intake, feed efficiency, and carcass characteristics of young Nellore heifers under different days on feed in the feedlot. Animals.

[15] Sanjorjo R.A., Tseten T., Kang M.-K., Kwon M., Kim S.-W. (2023). In pursuit of understanding the rumen microbiome. Fermentation.

[16] Yu Z., Yan M., Wang J. (2024). Rumen microbiome nutriomics: Harnessing omics technologies for enhanced understanding of rumen microbiome functions and ruminant nutrition. Anim. Nutr..

[17] Chen K., Shui Y., Deng M., Guo Y., Sun B., Liu G., Liu D., Li Y. (2024). Effects of different dietary energy levels on growth performance, meat quality and nutritional composition, rumen fermentation parameters, and rumen microbiota of fattening Angus steers. Front. Microbiol..

[18] Zhu Z., Zhang J., Shah A.M., Zhang Q., Bai B., Hao L. (2026). Production, Transport, and Metabolism of Volatile Fatty Acids in the Yak Rumen: Unraveling the Unique Mechanisms Underpinning High-Altitude Adaptation. Microorganisms.

[19] Zhu X., Liu B., Xiao J., Guo M., Zhao S., Hu M., Cui Y., Li D., Wang C., Ma S. (2022). Effects of different roughage diets on fattening performance, meat quality, fatty acid composition, and rumen microbe in steers. Front. Nutr..

[20] AFRC (1992). Nutritive requirements of ruminant animals: Protein. AFRC Technical Committee on Responses to Nutrients. Report No. 9. Proceedings of the Nutrition Abstracts and Reviews (Series B).

[21] AMSA (2015). American Meat Science Association Research Guidelines for Cookery, Sensory Evaluation, and Instrumental Tenderness Measurements of Meat.

[22] Wang H., Li H., Wu F., Qiu X., Yu Z., Niu W., He Y., Su H., Cao B. (2019). Effects of dietary energy on growth performance, rumen fermentation and bacterial community, and meat quality of Holstein-Friesians bulls slaughtered at different ages. Animals.

[23] Broderick G., Kang J. (1980). Automated simultaneous determination of ammonia and total amino acids in ruminal fluid and in vitro media. J. Dairy Sci..

[24] Kellogg D. (1969). Analysis of rumen fluid volatile fatty acids by chromatography with Porapak QS. J. Dairy Sci..

[25] Zhou X., Liu X., Liu M., Liu W., Xu J., Li Y. (2024). Comparative evaluation of 16S rRNA primer pairs in identifying nitrifying guilds in soils under long-term organic fertilization and water management. Front. Microbiol..

[26] Bolyen E., Rideout J.R., Dillon M.R., Bokulich N.A., Abnet C.C., Al-Ghalith G.A., Alexander H., Alm E.J., Arumugam M., Asnicar F. (2019). Reproducible, interactive, scalable and extensible microbiome data science using QIIME 2. Nat. Biotechnol..

[27] Rognes T., Flouri T., Nichols B., Quince C., Mahé F. (2016). VSEARCH: A versatile open source tool for metagenomics. PeerJ.

[28] Segata N., Izard J., Waldron L., Gevers D., Miropolsky L., Garrett W.S., Huttenhower C. (2011). Metagenomic biomarker discovery and explanation. Genome Biol..

[29] Wemheuer F., Taylor J.A., Daniel R., Johnston E., Meinicke P., Thomas T., Wemheuer B. (2020). Tax4Fun2: Prediction of habitat-specific functional profiles and functional redundancy based on 16S rRNA gene sequences. Environ. Microbiome.

[30] Bergman E. (1990). Energy contributions of volatile fatty acids from the gastrointestinal tract in various species. Physiol. Rev..

[31] Zhuang Y., Abdelsattar M.M., Fu Y., Zhang N., Chai J. (2024). Butyrate metabolism in rumen epithelium affected by host and diet regime through regulating microbiota in a goat model. Anim. Nutr..

[32] Yi S., Dai D., Wu H., Chai S., Liu S., Meng Q., Zhou Z. (2022). Dietary concentrate-to-forage ratio affects rumen bacterial community composition and metabolome of yaks. Front. Nutr..

[33] Pang R., Xiao X., Mao T., Yu J., Huang L., Xu W., Li Y., Zhu W. (2023). The molecular mechanism of propionate-regulating gluconeogenesis in bovine hepatocytes. Anim. Biosci..

[34] Wang G., Zhu Y., Feng D., Yao J., Cao Y., Deng L. (2025). Hepatic gluconeogenesis and regulatory mechanisms in lactating ruminants: A literature review. Anim. Res. One Health.

[35] Llonch L., Castillejos L., Ferret A. (2020). Increasing the content of physically effective fiber in high-concentrate diets fed to beef heifers affects intake, sorting behavior, time spent ruminating, and rumen pH. J. Anim. Sci..

[36] Qiu X., Qin X., Chen L., Qiu Q., Wang H., Aziz ur Rahmanand M., Cao B., Su H. (2021). Effects of age and rice straw inclusion levels in the diet of Yiling cull cows on growth performance, meat quality, and antioxidant status of tissues. Animals.

[37] Khatri Y., Huff-Lonergan E. (2025). Eating quality of ribeye steaks from young bulls and steers is comparable: A study examining the effect of sex, frame size, and feed time on palatability attributes. Food Sci. Nutr..

[38] Song Z., Hwang I. (2023). Objective meat quality from quality grade and backfat thickness of Hanwoo steers. Food Sci. Anim. Resour..

[39] Zhang L., Liu S., Wang X., Wang H., Li S., Zhen Y., Zhang X. (2025). Effect of Harvesting Time on Starch Degradation in Rumen of Whole-Plant Corn and Its Silage. Fermentation.

[40] Rawal S., Kaur H., Bhathan S., Mittal D., Kaur G., Ali S.A., Kumar Yata V., Mohanty A.K., Lichtfouse E. (2024). Ruminant Gut Microbiota: Interplay, Implications, and Innovations for Sustainable Livestock Production. Sustainable Agriculture Reviews: Animal Biotechnology for Livestock Production 4.

[41] Wei W., Zhen Y., Wang Y., Shahzad K., Wang M. (2022). Advances of Rumen Functional Bacteria and the Application of Micro-Encapsulation Fermentation Technology in Ruminants: A Review. Fermentation.

[42] Peng W., Wang Y., Wei M., Liu S., Liu K., Xiao M., Zhang R., Wang Y., Zheng Y., Fang L. (2025). Effects of changes in rumen microbial adaptability on rumen fermentation and nutrient digestion in Horqin beef cattle during different seasons of grazing and supplementary feeding. BMC Microbiol..

[43] Chen H., Wang C., Huasai S., Chen A. (2021). Effects of dietary forage to concentrate ratio on nutrient digestibility, ruminal fermentation and rumen bacterial composition in Angus cows. Sci. Rep..

[44] Matchado M.S., Rühlemann M., Reitmeier S., Kacprowski T., Frost F., Haller D., Baumbach J., List M. (2024). On the limits of 16S rRNA gene-based metagenome prediction and functional profiling. Microb. Genom..

